# Family discussions and demographic factors influence adolescent’s knowledge and attitude towards organ donation after brain death: a questionnaire study

**DOI:** 10.1186/s12910-020-00499-x

**Published:** 2020-07-09

**Authors:** Vanessa Stadlbauer, Christoph Zink, Paul Likar, Michael Zink

**Affiliations:** 1 Department of Internal Medicine, Research Unit “Transplantation Research”, Division of Gastroenterology and Hepatology, Medical University of Graz, Auenbruggerplatz 15, 8036 Graz, Austria; 2 Department of Anaesthesiology and Intensive Care Medicine, Hospital of the Brothers of St. John of God, St. Veit an der Glan, Austria and Hospital of the Elisabethinen Klagenfurt, Klagenfurt, Austria

**Keywords:** Organ donation, Attitude, Adolescent, High school, Education

## Abstract

**Background:**

Knowledge and attitude towards organ donation are critical factors influencing organ donation rate. We aimed to assess the knowledge and attitude towards organ donation in adolescents in Austria and Switzerland.

**Methods:**

A paper-based survey was performed in two secondary schools (age range 11–20 years) in Austria and Switzerland. 354/400 surveys were sufficiently answered and analyzed.

**Results:**

Our study found that knowledge on organ donation is scarce in adolescents. Less than 60% of those surveyed thinks that a person is dead when declared brain dead. 84.6% would authorize organ donation after brain death for themselves, but only 69% would authorize organ donation after brain death for a close relative. 93.7% would accept a donor organ if they needed one. Family discussions, rather than school discussions, influenced knowledge on organ donation, the percentage of respondents who have a firm opinion on organ donation and the rate of declaration of this opinion. Age, gender, nationality and religion also influenced knowledge and attitude towards organ donation. Nearly one third of adolescents are of the opinion that selling non-vital organs should be legalized.

**Conclusion:**

Since having had family discussions, a potentially modifiable factor, was positively associated with knowledge and attitude towards organ donation, we postulate that educational programs stimulating family discussions on organ donation may be a promising strategy to increase knowledge.

## Background

The number of organ donations after brain death vary widely across countries, as exemplified by the Eurotransplant figures for 2018, which show an organ donation rate between 29.4/million population in Belgium and 11.3/million population in Germany [1]. Factors that influence organ donation rate are manifold: legislation, organization of the organ donation system, culture, religion, education and public opinion and trust in the donation system are all important factors influencing the rate of organ donation in a country [2–5]. Knowledge and education about organ donation are key factors for a positive attitude towards this process. Demographic factors such as age, gender, level of education, field of occupation and place of residence influence also knowledge and attitude. Younger age, female gender and higher knowledge level are factors positively associated with an increased willingness to donate organs [6]. We previously published for Austria that, besides gender and prior knowledge, religious and cultural norms also influence the opinion towards organ donation [7, 8]. The European Commission performed a survey about the opinion of the general public in the European Union in 2009 and found that there was a low level of support of organ donation in Austria, however, the level of family discussions and the willingness to donate increased from 2006 to 2009 [9]. Many studies have been performed to understand the knowledge and attitude of younger people, in most cases university students, towards organ donation after brain death [10]. It is known that level of knowledge, educational courses, value priorities, gender and ethnicity influence adolescent’s opinion and attitude towards organ donation after brain death [11–13]. In Austria, a presumed consent (also called opt-out) system is in place, where adolescents can decide above the age of 14 on their own whether they want to register for the organ donation objection register. In contrast, in Switzerland, an opt-in system requiring authorization by the donor (by registration in the national organ donation register) or authorization of the next of kin is in place. Adolescents above the age of 16 can register themselves in the Swiss national organ donation register. To enable adolescents to make such a decision in an informed manner, it is important to tailor educational programs to their needs. In a first step, it is therefore necessary to assess knowledge and attitude towards organ donation. By performing a literature review, we identified a knowledge gap on how adolescents in Austria and Switzerland think about organ donation after brain death. In this study, we examined the influence of education (e.g. family and school discussions), gender, religion and nationality on the opinion and attitude towards organ donation in a cohort of secondary school pupils in Austria and Switzerland to be able to tailor educational efforts to the needs of this group.

## Methods

A paper-based questionnaire in German (for original version and English translation, see supplementary materials 1 and 2) was developed based on previously structured questionnaires used by the authors [7, 8]. The questionnaire consisted of 3 pages, and contained a short introduction on organ donation, followed by 5 demographic questions (age, gender, nationality, religion, year at school) and 14 questions regarding knowledge and attitude towards organ donation (9 binary answer questions and 5 multi-answer multiple choice questions). The introductory text was adapted to the presumed knowledge of secondary school pupils. The questions were adapted to reflect the demographic; year of school replaced highest level of education and health care profession related questions were omitted. In addition to the previous questionnaires, we developed further questions regarding previous discussions within the family or at school, as well as personal reasons for or against organ donation. We assessed the attitude towards organ donation at different levels: we asked if the participants already had a firm opinion, if they had officially declared their opinion (e.g. by carrying an organ donation card or by having set up a patient provision) and we asked if they would in general authorize organ donation for themselves or close relatives. Face validity, feasibility and utility was tested by 10 people (medical and non-medical professionals unrelated to the study) before fielding the questionnaire. Internal consistency on those items that measure the same dimension was performed using Cronbach’s alpha and was found to be acceptable (alpha = 0.7). The questionnaire was distributed to adolescents between 11 and 20 years of age two secondary schools (one in Austria and one in Switzerland, chosen because of personal connections of the authors) and filled in at school under supervision of the responsible teacher. Use of this questionnaire was approved by the rectors of Lyceum Alpinum Zuoz (LAZ, CH) as well as Bundesgymnasium Tanzenberg (BT, Austria) respectively and by the department of education of the county Carinthia (A/0013-R/2019) as part of the high school thesis of Ch.Z. A separate approval by an ethics committee is not required according to the Austrian School Education Act. Answers were provided anonymously, and participants were instructed verbally before beginning that filling in the questionnaire acted as their consent to analysis and publications, and that they could withdraw consent at any time by stopping the questionnaire. Therefore, consent was implied upon completion of the questionnaire. No separate consent from parents or guardians was obtained.

Data from the paper questionnaires were transferred to an electronic database and record verification was performed by double entry of 10% of the questionnaires. Missing data were not replaced. SPSS V26 (IBM, Armonk, NY, USA) was used for analysis. Descriptive data are presented as absolute numbers, percentages or by median and 95% confidence interval (CI). First, univariate inter-group data analysis for previous family and school discussions, gender, nationality and religion were performed using independent sample t-test and chi-squared test. In addition, multivariate logistic regression was used to identify which of the factors identified by univariate analysis influenced the formation of a firm opinion towards organ donation. A *p* < 0.05 was considered statistically significant.

## Results

### Demographic characteristics of the study population

**Fig. 1:**
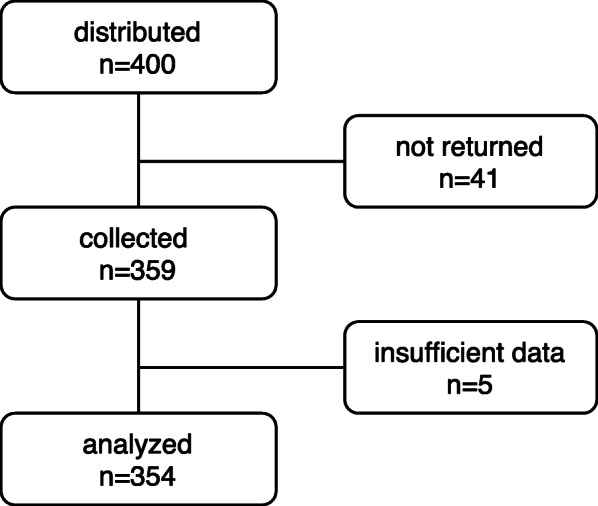
Study flow chart

Four hundred questionnaires (200 at LAZ and 200 at BT) were distributed between June and October 2019. Three hundred fifty-nine questionnaires were returned (89.8%), 5 of these were insufficiently answered (3 LAZ, 2 BT) and therefore excluded from analysis. 354 (88.5%) questionnaires were included in the analysis (Fig. 1). Religion was grouped into Christian (Roman Catholic and Protestant), atheist and other religions because these groups were previously described to influence knowledge and attitude towards organ donation and this grouping resulted in comparable sample sizes in our study. Nationality was grouped into Austria, Germany+Switzerland and others because of the different legal situation regarding organ donation in Austria compared to Switzerland. Germany was grouped together with Switzerland due to a comparable legal situation; due to the small sample size from Germany (*n* = 13, 3.7% of the cohort), it was not feasible to analyze this nationality separately. To account for the internationality of one of the schools, a group of “other” countries was necessary. Table 1 shows the demographic characteristics of the study cohort.

**Table 1:** Demographic characteristics of the participants

	*N* = 354
Age years median (95% CI)	15 (15;16)
Gender m/f n (%)	170/184 (48/52)
Year at school (years)	9 (9–10)
School type LAZ/BT (%)	158/196 (44.6/54.4)
Nationality	
Austria n (%)	194 (54.8)
Germany and Switzerland n (%)	90 (25.4)
Others^a^ n (%)	70 (19.8)
Religion	
Christian n (%)	276 (78)
Atheist n (%)	61 (17.2)
Others^b^ n (%)	17 (4.8)

^a^Aserbaidschan, Australia, Belgium, Bulgaria, Bosnia-Herzegovina, Brazil, Estland, Lichtenstein, Croatia, Italy, Japan, China, Columbia, Kosovo, Mexico, the Netherlands, Portugal, Romania, Russia, Taiwan, Turkey, United Kingdom. Ukraine, USA, Usbekistan, Vietnam

^b^Islam, Buddhism, Judaism

### Opinion of adolescents regarding organ donation

Less than half (45.2%) of the respondents stated that they have a firm opinion on organ donation and only 21.3% have officially declared this opinion (e.g. by carrying an organ donation card or by having set up a patient provision). Of those who already officially declared their opinion, 81.3% declared that they authorized organ donation, whereas the remaining 18.7% filed an objection. When asked for their opinion, 84.6% would authorize organ donation after brain death for themselves, but only 69% would authorize organ donation after brain death for a close relative. 93.7% would accept a donor organ in case they would need one. 29% of adolescents had discussed organ donation in their families whereas only 17.3% already had discussions at school.

### Knowledge of adolescents about organ donation

Only 59.8% of the participants of our survey believe that a person is really dead when he/she is declared brain dead. When adolescents were asked which legal options for organ donation they know (multiple answers possible), 87.7 and 80.9% of the responses were correct by stating that volunteer living donation and donation after brain death are legal. However, 11.1 and 8.5% were of the opinion that buying an organ from either a brain dead donor or a living donor is legal. Adolescents were also asked, which legal possibilities to authorize organ donation they know (multiple answers possible). More than half of the respondents (54.7%) named opt-in (e.g. organ donation card), 27.2% authorization by next-of kin and 18.3% opt-out. 37% stated that they do not know which possibilities exist. Only 5.3% of the participants named all 3 correct answers. Interestingly, 29.8% stated that, in their opinion, it should be legal to sell non-vital organs. Knowledge on political discussions to change existing organ donation laws was scarce; 88.7% stated that they have not heard about any of these efforts, 3.7% thought that such efforts take place in Austria (which is not true), 4.5% in Germany and 5.4% in Switzerland (both true).

### Attitude towards organ donation

When asked for their personal arguments to donate organs (multiple answers possible), 74.9% named the wish to help, 73.4% the possibility to receive an organ as reasons, 33.1% would take pride in helping somebody else, 23.9% think that organ donation would give death a purpose, and 4.6% would donate for religious reasons. Interestingly, when asked within this context, only 8.9% stated that they would not donate organs at all. When the entire cohort was next asked for their personal arguments against organ donation (multiple answers possible), 40.3% did not want to make a decision yet, 36% were afraid of abuse through organ trafficking, 29.5% were afraid that in case of authorization of organ donation doctors would not do everything to save their lives, 31.5% were afraid that they might not be dead after declaration of brain death, 10.4% generally did not want to donate, 8.8% thought that organ donation disturbs the peace of death or disfigures the body and 5.8% had religious reasons.

### Influence of previous discussions about organ donation on knowledge and attitude of adolescents towards organ donation

**Fig. 2:**
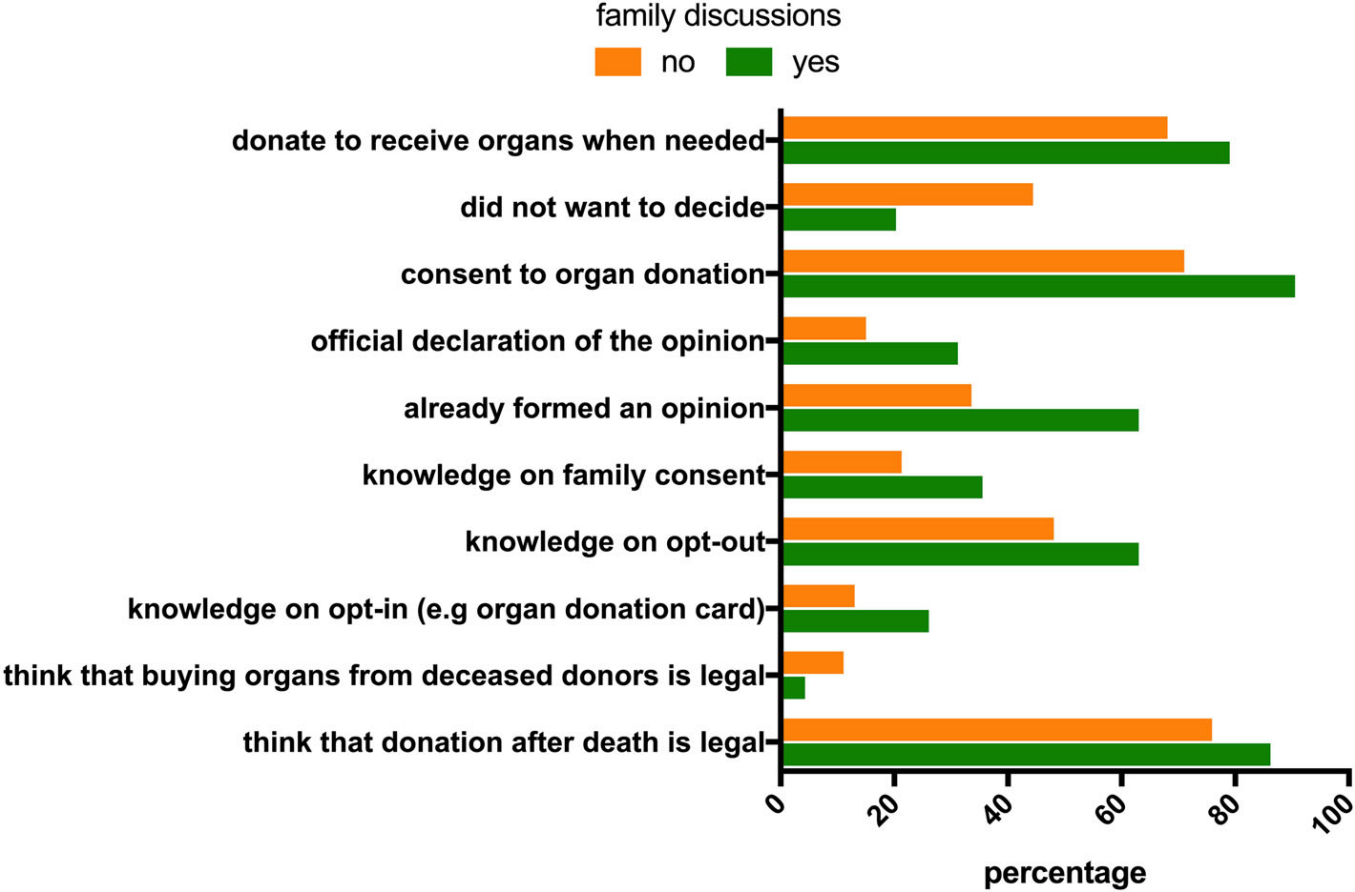
Significantly different answers depending on whether adolescents had (yes) or had not (no) discussed organ donation with their families

Previous family discussions on organ donation influenced the level of knowledge: 86.2% versus 75.9% of those without family discussions knew that donation after death is legal (*p* = 0.02) and 4.3% versus 11.1% of those without family discussions thought that selling organs from brain dead donors is legal (*p* = 0.03). Also, significantly more participants who had family discussions knew that opt-out (26.1% versus 13%, *p* = 0.03), opt-in (63% versus 48.1%, *p* = 0.06) or authorization by the family (35.5% versus 21.3%, *p* = 0.03) are legal options for organ donation. Previous school discussions on organ donation were interestingly associated with a higher percentage of participants thinking (wrongly) that buying organs from brain dead donors is legal (16.4% versus 6.8%, *p* = 0.02) (Fig. 2). No other answers regarding knowledge of organ donation were influenced by school discussions. Neither family nor school discussions influenced the knowledge on attempts to change the legislation in different countries.

A significantly higher percentage of adolescents who had family discussions on organ donation had a firm opinion regarding organ donation (63% versus 33.6% in those who had no family discussions, *p* < 0.0001). Similarly, family discussions on organ donation was associated with a higher percentage of official declaration of the opinion (31.2% versus 15.0% in those who had no family discussions, *p* < 0.001), and of authorization of organ donation (90.5% versus 71.9% in those who had no family discussions, *p* = 0.002) (Fig. 2). Accordingly, having a firm opinion on organ donation led to a considerably higher percentage of official declaration of the opinion (40.9% versus 5.2% in those who had no firm opinion, *p* < 0.001) (Fig. 2). School discussions did not influence any of these answers. Neither family discussions not school discussions on organ donation influenced the percentage of those who would accept an organ for themselves, who would allow the donation of their own organs after brain death or who would allow donation of their relatives’ organs after brain death. Also, the opinion on legalization to sell non-vital organs was not influenced by family or school discussions on organ donation. When asked for their personal arguments for and against organ donation, significantly fewer of those who had family discussions said that they do not want to decide (20.3% versus 44.4%, *p* < 0.001) and significantly more said that they would donate because they also would like to receive an organ in case they need one (79% versus 68.1%, *p* = 0.028). School discussions did not influence personal arguments for or against organ donation (Fig. 2).

### Influence of demographic factors on adolescents’ attitude and knowledge towards organ donation

**Fig. 3:**
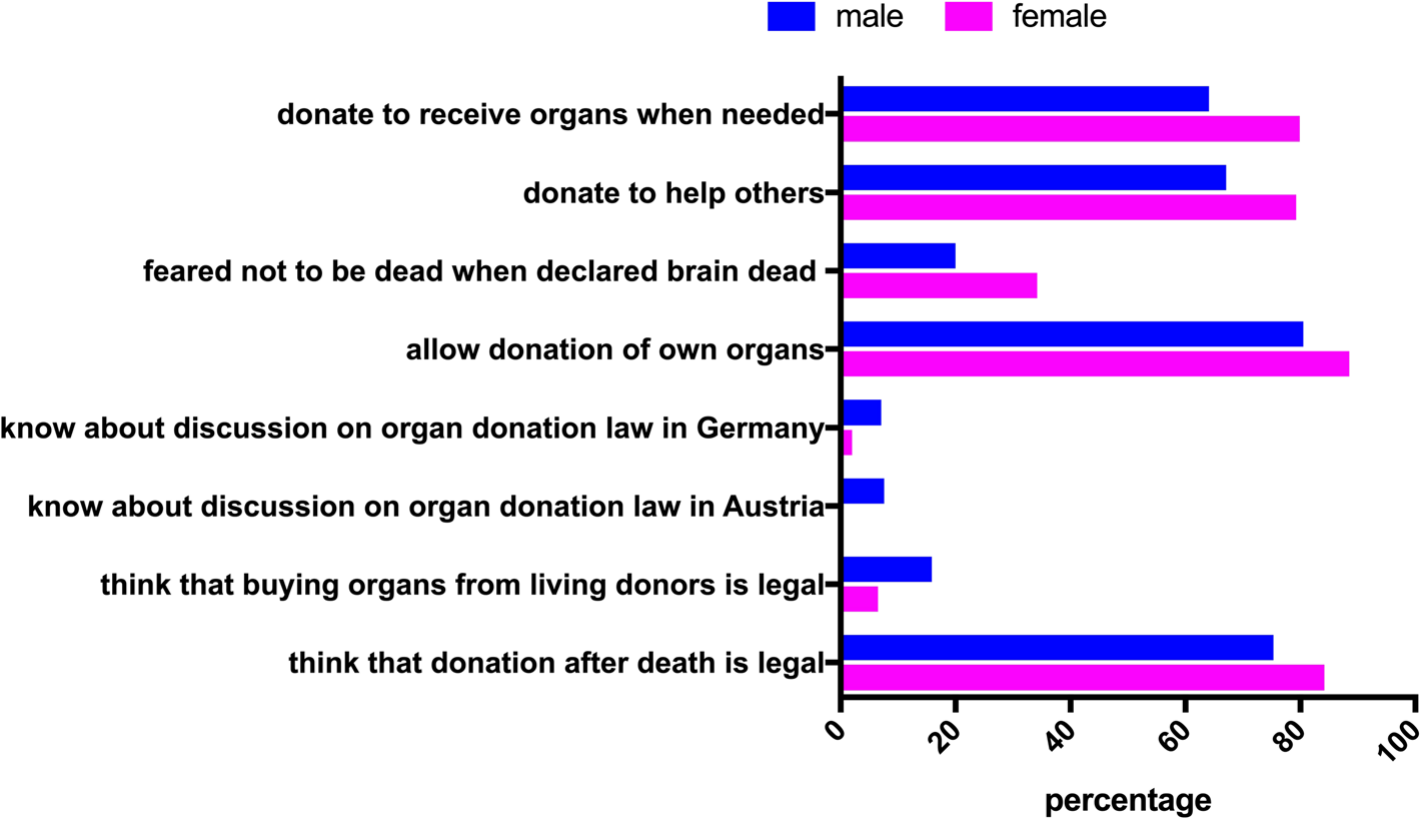
Significantly different answers between female and male participants

Participants who had a firm opinion on organ donation were on average 1 year older than those who had no firm opinion (16 (16;17) versus 15 (15;16) years, *p* = 0.001). Gender significantly influenced knowledge about organ donation; 84.2% of female adolescents knew that donation after death is legal, compared to 75.3% of the male participants. Fewer females also thought that buying organs from living donors is legal (6.5% versus 15.9%, *p* = 0.04). No female participant stated that they had heard about discussions to change the organ donation law in Austria (which is true), as opposed to 7.6% of male participants (*p* = 0.001), but fewer female participants stated that they had heard about these discussions in Germany, where such discussions took place (2.2% versus 7.1% in male participants, *p* = 0.038). Gender also significantly influenced the attitude towards organ donation; 88.5% of females would allow organ donation after their own brain death whereas only 80.4% of male adolescents would only allow it (*p* = 0.04). Regarding their personal reasons, more female adolescents feared that they would not be really dead when declared brain dead (34.2% versus 20% of male participants, *p* = 0.003). Significantly more females would donate organs to help others (79.3% versus 67.1%, *p* = 0.01), and because they also would like to receive an organ in case they need one (79.9% versus 64.1%, *p* = 0.001) (Fig. 3).

Regarding nationality, participants from 29 nations took part in the survey, due to the internationality of one of the two schools (LAZ, Switzerland), whereas in the Austrian school BT, people from only 6 nations participated. Therefore, nationality was grouped into Austria, Germany+Switzerland and “other countries” as described above.

91.2% of Austrian participants knew that volunteer living donation is legal, compared to 85.6% in participants from Germany+Switzerland and 75.7% in participants from other countries (*p* = 0.004). Similarly, Austrian participants knew in 87.6% that donation after death is legal, compared to 75.6% in participants from Germany+Switzerland and 64.3% in participants from other countries (*p* = 0.001). On the other hand, 11.1% of the participants from Germany+Switzerland and 27.1% in participants from other countries thought that selling organs from living donors is legal, compared to 5.2% of Austrian participants (*p* = 0.001). Results were comparable for selling organs from brain dead donors (10.0, 18.6 and 4.1% respectively, *p* = 0.001). Nearly half of the participants from other nations (45.7%) compared to 24.7% of the Austrian and 28.4% of the Germany+Switzerland participants thought that selling organs should be legalized. More participants from Austria (58.8%) and Germany+Switzerland (54.4%) knew that an organ donation card is an option to declare the will regarding organ donation as compared to participants from other nations (40%) (*p* = 0.026). More participants from Germany+Switzerland (7.8%) and other nations (10%) knew that a change in legislation was discussed in Switzerland compared to Austrian participants (2.6%) (*p* = 0.03). Fewer Austrian participants stated that they had a firm opinion (38.9%) compared to Germany+Switzerland (52.8%) and other nations (52.2%) (*p* = 0.03). This resulted in less frequent declaration of their opinion in Austrians (12.4%) compared to Germany+Switzerland (24.7%) and other nations (41.4%) (*p* = 0.0001). This might be influenced by the fact that Austrian participants were significantly younger (1 year) than participants from Germany+Switzerland and other nations (15 versus 16 years, *p* < 0.0001).

**Fig. 4:**
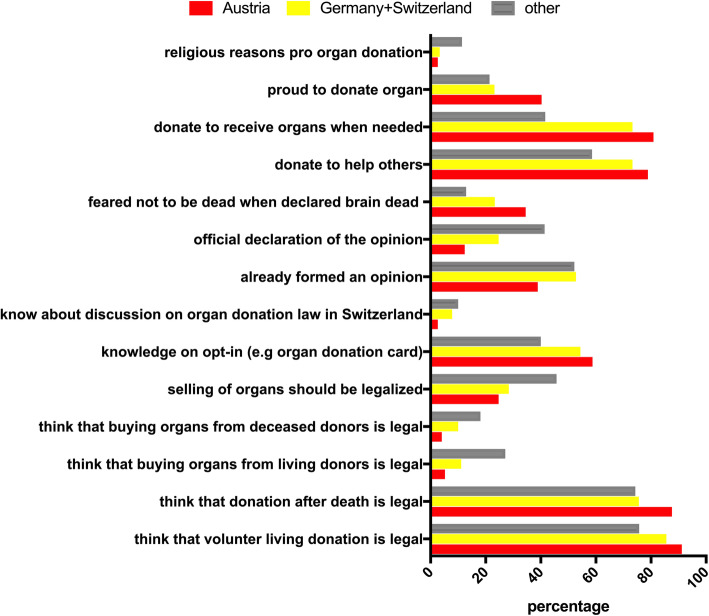
Significantly different answers between different nations

Significantly more Austrian participants (34.5%) named the fear of not being dead when declared brain dead as an argument against organ donation compared to Germany+Switzerland (23.3%) and other nations (12.9%) (*p* = 0.001). Austrians (78.9%) and participants from Germany+Switzerland (73.3%) would significantly more often donate organs to help others compared to other nations (58.6%) (*p* = 0.004). Significantly more Austrians (80.9%) and participants from Germany+Switzerland (73.3%) would also donate organs because they also would like to receive an organ in case they need one compared to other nations (47.1%) (*p* = 0.0001). For Austrians (40.7%), being proud to help others was more frequently a positive argument for organ donation compared to Germany+Switzerland (23.3%) and other nations (21.4%) (*p* = 0.001). Religious reasons were more frequently a positive argument for other nations (11.4%) compared to Austria (2.6%) and Germany+Switzerland (3.3%) (Fig. 4).

**Fig. 5:**
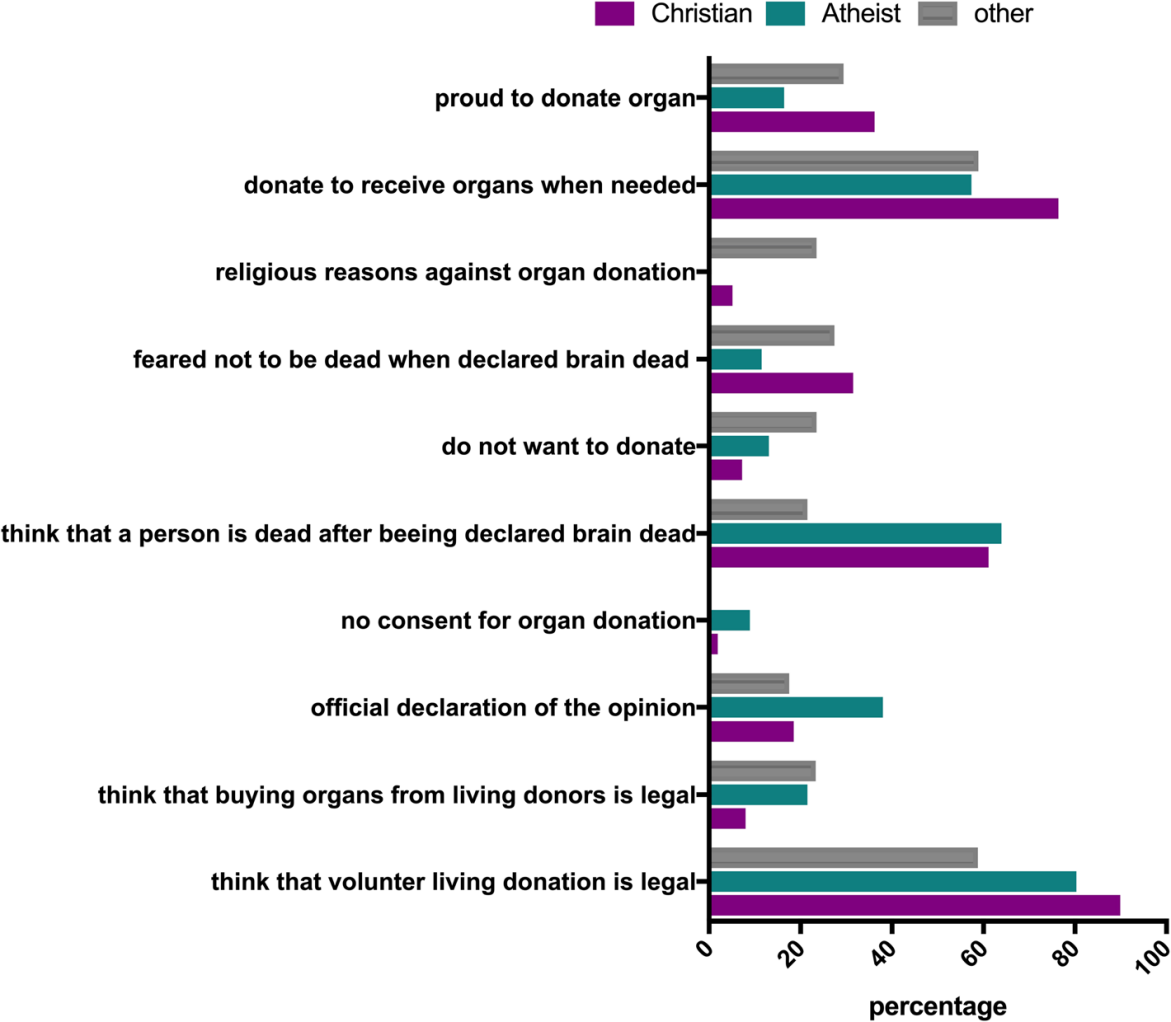
Significantly different answers between different religions

Religion also significantly influenced knowledge and attitude towards organ donation. Religion was grouped into Christian, atheist and “other religions” as described above. More Christians (89.9%) and atheists (80.3%) compared to participants with other religions (58.8%) knew that volunteer living donation is legal (*p* = 0.0001). More Atheists (21.3%) and participants with other religions (23.5%) compared to Christians (8%) thought that selling organs from living donors is legal. Declaration of an opinion regarding organ donation was more common in atheists (35%) versus Christians (18.5% and other religions 17.5%, *p* = 0.02). Atheists also opposed against organ donation more often (9.8%), compared to 2.9% of Christians and none of the participants with other religions (*p* = 0.03). 61.1% of Christians and 63.9% of atheists believed that a person is dead after declaration of brain death, whereas only 23.5% of those with other religions believe this (*p* = 0.007). The argument of not wanting to donate was more common amongst atheists (13.1%) and participants with other religions (23.5%) more frequently than Christians (7.2%) (*p* = 0.04), whereas Christians (31.5%) more often feared that they would not be really dead when declared brain dead compared to atheists (11.5%) and other religions (27.4%). Religious reasons as an argument against organ donations was more common amongst participants with other religions (23.5%) compared to Christians (5.1%) and atheists (0%) (*p* < 0.0001). Significantly more Christians (76.4%) would donate an organ because they also would like to receive an organ in case they need one compared to atheists (57.4%) and other religions (58.9%) (*p* = 0.005). For Christians (36.2%) and other religions (29.4%), being proud to help others was also more frequently a positive argument for organ donation compared to atheists (16.4%) (*p* = 0.01) (Fig. 5).

### Multivariate analysis

To understand which factors influenced participants’ firm opinions, we performed multivariate logistic regression analysis. We included age, nation and family discussions because these variables were significant on univariate analysis. Age (*p* = 0.003, odds ratio 1.21) and family discussions (*p* < 0.0001, odds ratio 3.40) remained significant explanatory variables for having a firm opinion on organ donation. Additionally, we also analyzed factors that influenced if adolescents would allow organ donation for themselves after brain death. We included age and gender in the model, and only gender (*p* = 0.043, odds ratio 1.87) remained as significant explanatory variable for allowing organ donation after brain death.

## Discussion

This survey studies factors that influence knowledge and attitude towards organ donation in adolescents from secondary schools in Austria and Switzerland. It was our aim to understand which factors influence the knowledge and attitude of adolescents towards organ donation to be able to tailor educational efforts to the needs of this group. Our survey shows that knowledge on organ donation has room for improvement in adolescents. The majority of those surveyed would allow organs to be donated after their death; less would allow donation for close relatives, but almost all participants would accept an organ for themselves. Less than half of those surveyed had a firm opinion on organ donation, and this opinion was influenced by age and whether the participants had already discussed organ donation within their families.

More than 40% of participants do not believe that a person who is declared brain dead is really dead, although the definition of brain death was given in the introductory text of the questionnaire. Our questionnaire was unfortunately not designed to find out whether these participants disagree or are unsure about equating brain death with a biological understanding or definition of death. This finding, however, suggests that information on the brain death concept should be included in public education.

15.4% of participants would not allow donation of organs from a close relative but would allow donation of their own organs after death. This group of participants had significantly less family discussions on organ donation. This difference is of relevance, especially in legal systems where family authorization or refusal plays a major role in the decision process for organ donation. For families, the burden of decision making in a situation of personal grief is high [14]. Several modifiable but also non-modifiable factors may influence this decision and special skills of the requester are necessary to prevent unnecessary loss of organs [15–17]. Besides the legal situation, the practice in a country may also influence the role of family decisions in the organ donation process [17]. Systems that do not delegate the burden of this decision to the grieving families may, in our opinion, provide a better framework to increase organ donation rate; however, this topic is heavily discussed worldwide and a change in the system has a huge legal, ethical and practical impact. Due to the diversity of the systems and the complexity of the organ donation process, the legal situation and the potential burden of decision for families are not the sole factors explaining varying organ donation rates. Systematic reviews suggest that countries with opt-out systems have higher rates of organ donations after brain death [18], but others showed that there are no differences between opt-in and opt-out systems [19, 20] depending on the studies and countries included in the analysis. There are indeed examples of highly successful countries with an opt-in system, such as the USA; however, switching from opt-out to opt-in does not necessarily result in a sustainable increase of organ donation rate [21, 22].

9.1% of the participants who would accept an organ for themselves stated that they would not donate their own organs. This mismatch, termed “free-riding” in literature, has been discussed ethically for decades [23] and has led to policy changes, for example in Israel, where holding an organ donor card leads to a preferred status in organ allocation in case a transplantation is needed as a way of compensation for organ donation [24].

Our results support the notion that in adolescents, the influence of the opinion of the family is of high relevance. Several studies already looked at adolescents’ knowledge and attitude towards organ donation in different regions of the world and found that the opinion of the family is an important factor. Adolescents from the USA, who did not want to donate organs and those who were undecided were less likely to have discussed their decision with parents than were those who wanted to donate [11, 25]. In the Netherlands, knowledge on organ donation in adolescents was positively associated with intent to register as an organ donor [12]. Our data support and expand this knowledge, since we could show that having discussed organ donation with the family, as well as age are the strongest factors that determine whether adolescents have already a firm opinion on organ donation or not. Family discussions and having a firm opinion both positively influenced the rate of official declaration of their opinion, and the majority of those who declared their opinion had a positive opinion (81.3%). From our data, we can conclude that it may be desirable to raise the number of adolescents who have a firm opinion on organ donation by supporting discussion in families. This is supported by data from Spain, a country with a high organ donation rate, where secondary school pupils received information about organ donation from family in 46% of cases, and positive information from the family was associated with a positive opinion towards organ donation [26]. Information from school was the source of information in 38% of cases, and school information did not positively influence the opinion on organ donation [26]. In our study, only 17.3% of the participants stated that they had already discussed organ donation at school. This figure is also comparable to data from Spain, where only 16% of teachers declared that they provide information on organ donation to pupils [27]. School discussions would also rely on the opinion and attitude of teachers. A survey among secondary school teachers showed that only half of the respondents believed that it would be appropriate to introduce an educational program on organ donation at school, indicating the need for education of teachers [28]. Classroom education has been shown to be effective in raising knowledge on organ donation and positively influencing the opinion in several studies in different countries [29–33], and may also stimulate family discussions and thereby indirectly influence organ donation rates [34].

Therefore, educational programs that target the general public and stimulate discussion on the topic within families seem promising. The question then arises on which educational efforts should be made to improve organ donation rates. Meta-analyses on campaigns and mass media exposure to educate the general public concluded that positive news on organ donation and educational campaigns irrespective of the scale (individual efforts, small scale workshops or national campaigns) are able to increase organ donation rates; however, the effect of public education campaigns is modest, with only an average 5% gain in organ donation rates. Negative mass media exposure, however, can drastically decrease organ donation rates, possibly for prolonged periods of time [35]. The role of social media in education about organ donation has to be viewed with caution. When Facebook allowed members in several states to add “organ donor” to their profile, a huge increase in people registering as organ donor was observed, but only for a few days. This effort was also criticized for not providing enough information for people to make an informed decision [36, 37]. We are of the opinion that repeated and transparent information using different channels (television, radio broadcasting, newspaper, social media etc.) may be a useful strategy to increase the exposure of the general public to this topic and stimulate family discussions.

Demographic factors such as age, gender, religion and nationality influenced knowledge and attitude towards organ donation in our study. This was expected from our own data in students and health care professionals [7, 8] and also from other previous studies in adolescents [11, 12]. Although these factors are unmodifiable, our data give an indication who should be the target groups of specific interventions. Our data show that a 1 year age difference during secondary school already significantly influences the likelihood of having a firm opinion on organ donation. Female respondents were better educated and more positive towards organ donation in our survey. Regarding nationality, differences in knowledge were observed, however, this may be biased by the fact that we only included two schools. The declaration of being an atheist was negatively associated with authorization of organ donation and religion and influenced the distribution of personal pro and con arguments.

An interesting finding in our survey was that nearly 1/3 of the respondents was of the opinion that selling organs should be legalized. The ethical acceptance of incentives or compensation for organ donation seems to be broad in younger adults [11, 38], despite clear international regulations that prohibit selling organs [39, 40]. Romanian health care professionals agree with the idea of compensation for organ donation, or even a free market for donor organs at a much higher percentage (81%) than in the general public (51%) [41]. Australian transplant physicians, however, think that direct financial incentives are morally questionable, but they would support removing financial disincentives in living donation [42]. Despite broad consensus against payments from authorities, the appropriateness of financial incentives for organs is clearly a matter of debate for many, especially younger, people.

Our study has some limitations: only two schools were included, which were chosen because of personal connections of the authors, therefore the complete picture on adolescents’ views on organ donation in Austria and Switzerland may not have been captured. However, despite personal connections, no additional educational efforts took place in these schools that would have biased the results. There was some heterogeneity in demographic characteristics, especially regarding the mixture of nationalities between the two schools; therefore, it is difficult to distinguish between effects of nationality and age and effects of the school visited. To strengthen these findings, future studies would include more schools to represent a more diverse population, as well as larger cohorts to distinguish between the effects of the demographics of the population surveyed.

## Conclusion

In conclusion, our study provides interesting insights into the knowledge and attitude of adolescents towards organ donation in Austria and Switzerland. In our small pilot study, despite scarce knowledge on organ donation, more than 4/5 of adolescents between the ages of 11–20 would donate their own organs. Since we found that family discussions were the most prominent modifiable factor that influences knowledge and attitude towards organ donation in adolescents, our data may suggest that targeted public education programs to stimulate family discussions can be a promising strategy to increase the number of adolescents who have a firm opinion on organ donations. From our pilot study, this seems to be the prerequisite to official declaration of the opinion. Once adolescents have made up or declared their opinion, most have a favorable donation intention. The association between having had family discussions and the willingness to authorize organ donation may be a topic for further research. These results can be of relevance for stake holders involved in developing strategies to improve organ donation rates.

## Supplementary information

**Additional file 1.** German version of the survey.

**Additional file 2.** English version of the survey.

## Data Availability

The datasets generated and analyzed during the current study are available in the OSF repository at https://osf.io/2es9f

## Abbreviations

LAZ: Lyceum Alpinum Zuoz
BT: Bundesgymnasium Tanzenberg
