# Supplementary material for: Family discussions and demographic factors influence adolescent’s knowledge and attitude towards organ donation after brain death: a questionnaire study
Source: BMC Med Ethics. 2020 Jul 9;21:57. doi: 10.1186/s12910-020-00499-x (PMC7346659; doi:10.1186/s12910-020-00499-x)
Supplement: Supplementary file 1 — Additional file 1. German version of the survey. [file 12910_2020_499_MOESM1_ESM.docx]

**Umfrage zur Organspende**

Einige lebensbedrohliche Erkrankungen können nur mittels einer Organtransplantation behandelt werden. Diese Erkrankungen können jeden von uns treffen. Trotz dieser dringenden Notwendigkeit beobachtet man in einigen Ländern eine sehr unterschiedliche Bereitschaft, Organe nach dem Tod zu spenden. Deshalb gibt es derzeit Bestrebungen die gesetzlichen und organisatorischen Voraussetzungen in Ländern mit niedriger Organspenderate (z.B. Deutschland), mittlerer Rate (z.B. Schweiz) an die Gegebenheiten in Ländern mit hoher Rate (z.B. Österreich, Spanien) anzupassen. Diese Umfrage hat den Zweck die Meinung von Jugendlichen und jungen Erwachsenen als zukünftige Leistungs- und Entscheidungsträger zu untersuchen. Die Umfrage erfolgt anonym. Die Daten werden anonym elektronisch verarbeitet und in aggregierter Form in meiner Maturaarbeit und eventuell auch wissenschaftlich veröffentlicht.

**Wissen zur Organspende:**

Grundsätzlich kann man entweder Lebendspender (z.B.: Wenn man ein enges Verhältnis zum Empfänger des Organs hat) oder postmortaler (=nach dem Tod) Organspender werden. In Mitteleuropa muss man den Hirntod erlitten haben um als tot zu gelten und somit Organspender zu werden. Wenn sämtliche Funktionen des gesamten Gehirns irreversibel erloschen sind, sind die Voraussetzungen für den Hirntod und damit für den Tod erfüllt. Eine Organspende kommt in Frage, wenn ein oder mehrere Organe des Verstorbenen noch funktionieren. Der Kreislauf und die Beatmung, wird mit medikamentöser beziehungsweise apparativer Unterstützung aufrechterhalten.

**Meinung zur Organspende**

1. **Altersangabe in Jahren :** ___ Jahre
2. **Geschlecht: □** männlich **□** weiblich
3. **Nationalität:**
4. **Religionszugehörigkeit:**
5. **Schulstufe:** __. Schulstufe
6. **Ist für Sie ein Mensch mit Hirntod tot?**

Ja Nein

1. **Wurde in Ihrer Familie schon über Organspende gesprochen?**

Ja Nein

1. **Wurde in Ihrer Schule schon über Organspende gesprochen?**

Ja Nein

1. **Haben Sie sich bisher schon eine feste Meinung zur Organspende bilden können?**

Ja Nein

1. **Haben Sie Ihren Willen für oder gegen die Organspende bereits bekanntgegeben? (Organspendeausweis, Patientenverfügung, ...)?**

Ja

Wenn Ja: ich habe mich **für** die Organspende entschieden

ich habe mich **gegen** die Organspende entschieden

Nein

1. **Wenn man, um Ihr Leben zu retten bei Ihnen eine Transplantation durchführen müsste, würden Sie dafür ein Organ eines Verstorbenen annehmen?**

Ja Nein

1. **Wenn Sie aufgrund eines Unfalls oder einer Erkrankung Hirntod wären, würden Sie erlauben, dass Ihre Organe für die Rettung des Lebens anderer verwendet werden?**

Ja Nein

1. **Wenn aufgrund eines Unfalls oder einer Erkrankung ein naher Angehöriger Hirntod wäre, würden Sie befürworten, dass seine Organe für die Rettung des Lebens anderer Menschen verwendet werden?**

Ja Nein

1. **Welche legalen Möglichkeiten gibt es, um ein Organ zu bekommen?**

Freiwillige Organspende eines lebenden Menschen (Niere, Leber-Teil, Lungensegment)

Durch die Organspende eines Verstorbenen

Kaufen eines Organs (z.B.: Herz, Leber, …) eines Verstorbenen

Kaufen eines Organs (z.B.: Niere) von Lebenden

1. **Welche Gründe sprechen für Sie persönlich gegen eine Organspende?**

Ich fürchte den Missbrauch durch Organhandel

Ich will mich noch nicht entscheiden

Ich möchte kein Organ spenden

Ich habe Angst, dass die Ärzte nicht mehr alles unternehmen, um mein Leben zu retten,

wenn ich ein Organspender bin

Ich habe Angst, dass ich nach dem festgestellten Hirntod noch nicht Tod bin

Eine Organspende stört die Totenruhe

Eine Organspende entstellt meinen Körper

Religiöse Gründe

1. **Warum sind Sie bereit Ihre Organe nach dem Tod zu spenden?**

Ich möchte anderen helfen

Durch die Organspende hat mein Tod einen Sinn

Ich wäre auch selbst froh ein Organ zu erhalten, wenn ich eines bräuchte

Es würde mich stolz machen durch die Organspende anderen zu helfen

Aus religiösen Gründen

Trifft alles nicht zu —Ich will kein Organspender sein

1. **Welche Möglichkeiten der Zustimmung oder Ablehnung gibt es?**

Widerspruchslösung (= Wer nicht aktiv widerspricht ist automatisch Organspender)

Einwilligung durch einen Organspendeausweis

Einwilligung durch Zustimmung der Angehörigen

Ich weiß es nicht.

1. **Sollte man Organe, die nicht lebensnotwendig sind, beispielsweise eine Niere, wenn man noch lebt verkaufen können?**

Ja

Nein

1. **Wissen Sie ob in einem der folgenden Ländern Bemühungen (z.B. Volksinitiative, parlamentarische Gesetzesänderung) zur Änderung der Gesetze gemacht werden?**

Österreich

Deutschland

Schweiz

Nein, ich weiß es nicht.

**Vielen Dank für Ihre Unterstützung!**
