# Supplementary material for: Family discussions and demographic factors influence adolescent’s knowledge and attitude towards organ donation after brain death: a questionnaire study
Source: BMC Med Ethics. 2020 Jul 9;21:57. doi: 10.1186/s12910-020-00499-x (PMC7346659; doi:10.1186/s12910-020-00499-x)
Supplement: Supplementary file 2 — Additional file 2. English version of the survey. [file 12910_2020_499_MOESM2_ESM.docx]

**Questionnaire on organ donation**

Some life-threatening diseases can only be treated with an organ transplant. These diseases can affect everybody. Despite this urgent need, there is a very different willingness to donate organs after death in some countries. Therefore, efforts are currently being made to adapt the legal and organizational requirements in countries with low organ donation rates (e.g. Germany), medium rates (e.g. Switzerland) to the conditions in countries with high rates (e.g. Austria, Spain). The purpose of this survey is to examine the opinions of adolescents as future key player and decision-makers. The survey is anonymous. The data will be processed electronically and published in an aggregated form.

**Knowledge about organ donation:**

In principle, you can either become a living donor (e.g. if you have a close relationship with the recipient of the organ) or postmortem (= after death). In Central Europe you have to have suffered brain death to be considered dead and thus to become an organ donor. When all functions of the entire brain have irreversibly gone, the conditions for brain death and thus for death are fulfilled. Organ donation is an option if one or more organs of the deceased are still functioning. The circulation and ventilation is maintained with medicinal or apparatus support.

**Opinion on organ donation**

1. **Age in years :** ___ years
2. **Sex: □** male **□** female
3. **nationality:**
4. **religion:**
5. **Grade:** __
6. **Is a person with brain death dead for you?**

Yes No

1. **Have you talked with your family about organ donation?**

Yes No

1. **Have you talked about organ donation at school?**

Yes No

1. **Have you been able to form an opinion on organ donation?**

Yes No

1. **Have you already declared you opinion on organ donation? (organ donation card, patients´provision, ...)?**

Yes

If yes: I decided to donate organs

I decided against organ donation

No

1. **If you had to have a transplant to save your life, would you take an organ from a deceased donor?**

Yes No

1. **If you were brain dead from an accident or illness, would you allow your organs to be used to save the lives of others?**

Yes No

1. **If your next of kin was declared brain dead as a result of an accident or illness, would you allow that his/her organs could be used to save other people's lives?**

Yes No

1. **What are the legal options for organ donation?**

volunteer living donation (kidney, part of the liver or part of the lung)

organ donation after death

buying an organ (e.g. heart, liver) from a deceased donor

buying an organ (e.g. kidney) from a living donor

1. **What are the reasons for you personally against organ donation?**

I fear organ trafficking abuse

I do not want to decide yet

I do not want to donate an organ

I am afraid that the doctors would not do everything to save my life,

when I am an organ donor

I am afraid that I am not dead after my brain death

Organ donation disrupts the dead rest

Organ donation disfigured my body

Religious reasons

**16. What are reasons to donate your organs after death?**

I want to help others

With organ donation, my death makes sense

I would also be happy to get an organ myself if I needed one

It would make me proud to help others through organ donation

For religious reasons

Nothing applies - I don't want to be an organ donor

**17. What options for consent or rejection towards organ donation do you know?**

Opt-out solution (=those who do not actively object are automatically organ donors)

Consent (Opt-in) with an organ donation card

Consent of relatives

I do not know

1. **Should a person be able to sell organs that are not essential to life, such as a kidney, while alive?**

Yes

No

**19. Do you know whether efforts were made to change the law in one of the following countries (e.g. popular initiative, parliamentary amendment to the law)?**

Austria

Germany

Switzerland

No, I do not know.

**Many thanks for your support!**
